# Supplementary material for: Nucleosome destabilization by nuclear non-coding RNAs
Source: Commun Biol. 2020 Feb 11;3:60. doi: 10.1038/s42003-020-0784-9 (PMC7012929; doi:10.1038/s42003-020-0784-9)
Supplement: Supplementary file 1 — Supplementary Information [file 42003_2020_784_MOESM1_ESM.pdf]

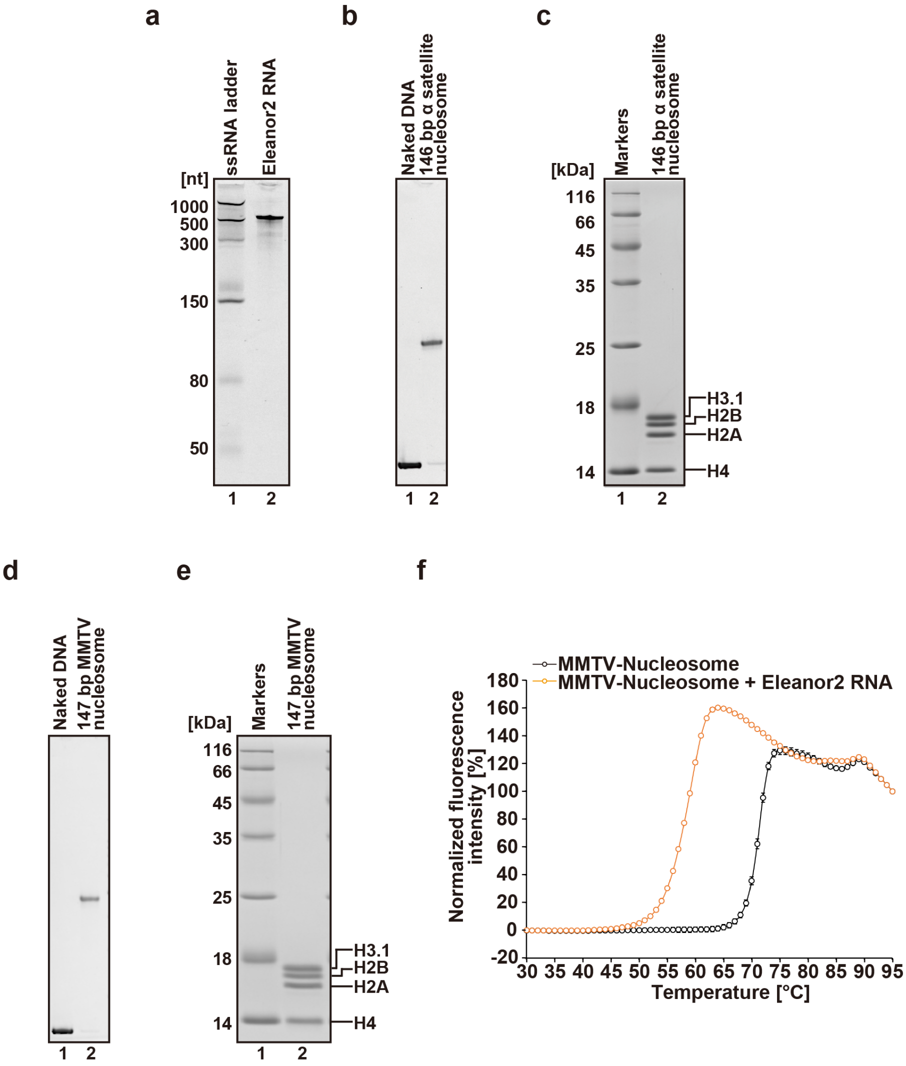

Supplementary Figure 1. Preparation of materials for in vitro nucleosome assays.
a, Purified Eleanor2 RNA transcribed in vitro was analyzed by 8% polyacrylamide/7 M urea denaturing gel electrophoresis with ethidium bromide staining. **b,** The reconstituted nucleosome with the 146 base-pair DNA, purified by a Prep Cell apparatus, was analyzed by 6% polyacrylamide gel electrophoresis with ethidium bromide staining. **c,** The histone contents of the nucleosome with the 146 base-pair DNA were analyzed by 18% SDS-polyacrylamide gel electrophoresis with CBB (Coomassie Brilliant Blue) staining. **d,** The reconstituted nucleosome with the 147 base-pair MMTV-DNA, purified by a Prep Cell apparatus, was analyzed by 6% polyacrylamide gel electrophoresis with ethidium bromide staining. **e,** The histone contents of the nucleosome with the 147 base-pair MMTV-DNA were analyzed by 18% SDS-polyacrylamide gel electrophoresis with CBB staining. **f,** Thermal stability curves of the MMTV-DNA nucleosome (1.25 μM) in the presence or absence of Eleanor2 RNA (1.25 μM). The fluorescence intensity was plotted against the temperature (from 30°C to 95°C). The means ± s.d. (n = 3) are shown. The uncropped gel images and the source data for the thermal stability assay are shown in Supplementary Figs. 5 and 9, respectively.

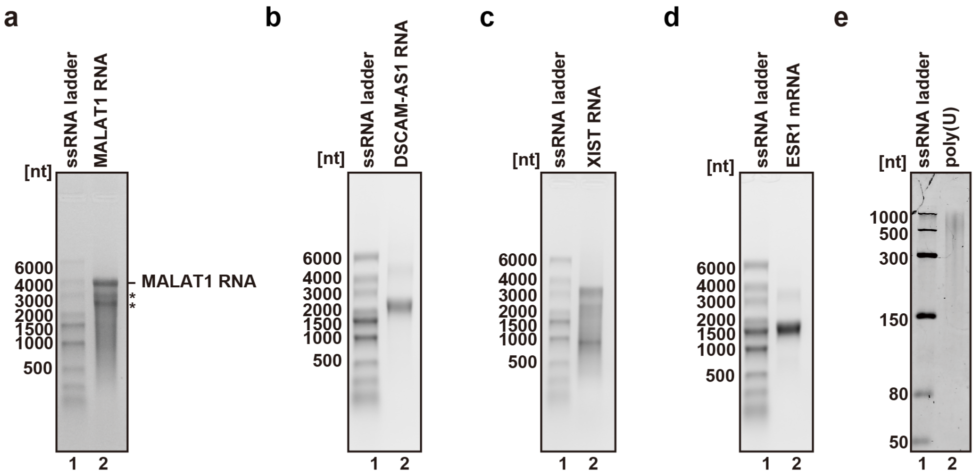

Supplementary Figure 2. Purification of various RNAs.

a, Purified MALAT1 RNA was analyzed on a 1% agarose gel containing 2.2 M formaldehyde with ethidium bromide staining. Bands with an asterisk and smeared bands are incomplete transcripts or degradation products. **b,** Purified DSCAM-AS1 RNA was analyzed on a 1% agarose gel containing 2.2 M formaldehyde with ethidium bromide staining. **c,** Purified XIST RNA was analyzed on a 1% agarose gel containing 2.2 M formaldehyde with ethidium bromide staining. **d,** Purified ESR1 mRNA was analyzed on a 1% agarose gel containing 2.2 M formaldehyde with ethidium bromide staining. **e,** Poly(U) was analyzed by 8% polyacrylamide/7 M urea denaturing gel electrophoresis with SYBR Gold staining. Poly(U) was not stained clearly by ethidium bromide, because of its unusual nature. The uncropped gel images are shown in Supplementary Fig. 5.

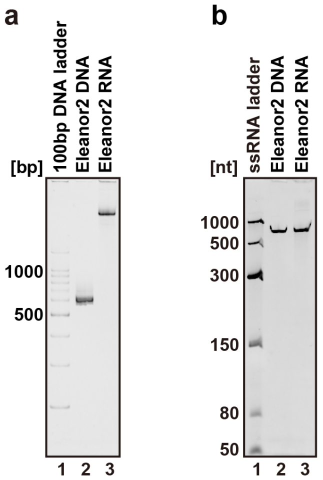

Supplementary Figure 3. Eleanor2 RNA is folded in vitro.
The migration distances of the Eleanor2 DNA and RNA are different in the non-denaturing 6% polyacrylamide gel, but are the same in the denaturing 6% polyacrylamide/7 M urea denaturing gel. Therefore, the Eleanor2 RNA should be folded in solution. **a,** Eleanor2 DNA and RNA were analyzed by non-denaturing 6% polyacrylamide gel electrophoresis with ethidium bromide staining. **b,** Eleanor2 DNA and RNA were analyzed by denaturing 6% polyacrylamide/7 M urea denaturing gel electrophoresis with ethidium bromide staining. The uncropped gel images are shown in Supplementary Fig. 5.

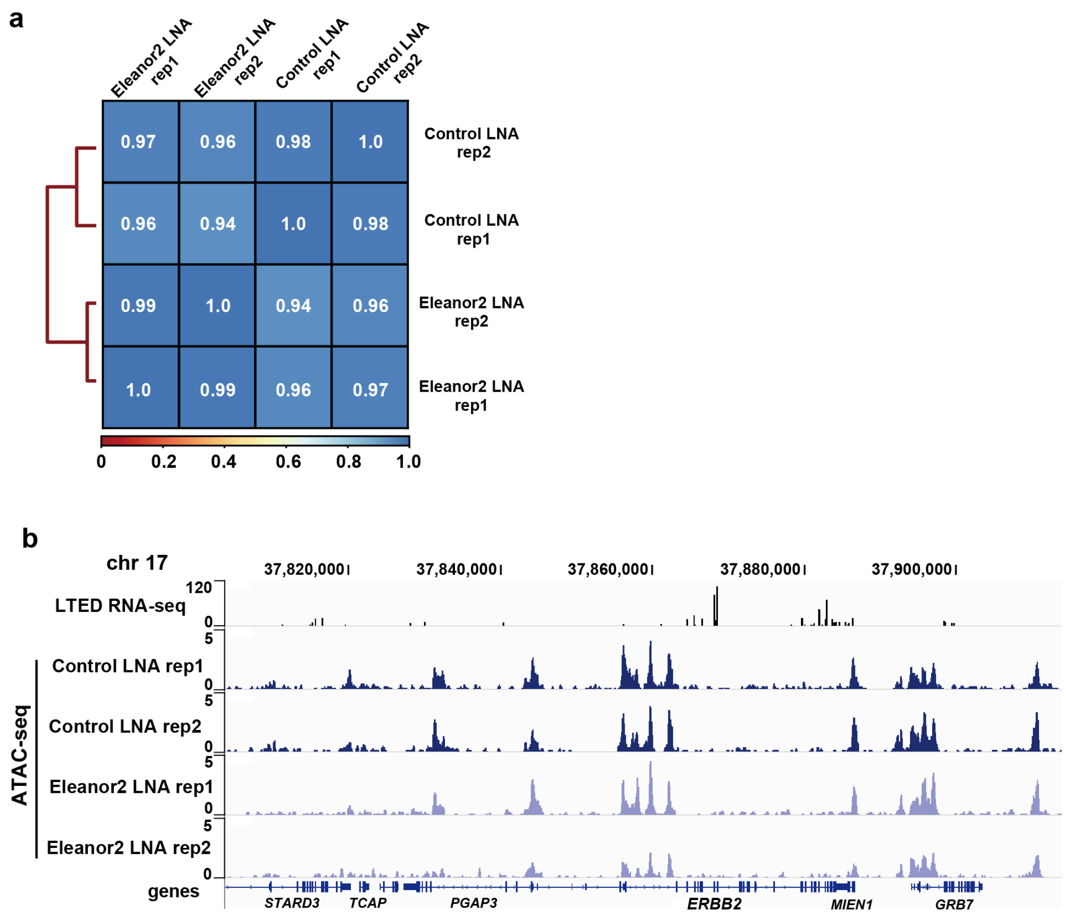

Supplementary Figure 4. Eleanor2 depletion did not affect the chromatin accessibility in other regions.

a, Reproducibility of the replicated ATAC-seq experiments (rep1 and rep2) using the LTED cells treated with either the Control or Eleanor2 LNA. The heatmap shows Pearson's correlation coefficients. **b,** Overview of the ATAC-seq profiles at the *ERBB2* locus and its neighboring region. Eleanor2 depletion did not influence the chromatin accessibility at the *ERBB2* locus.

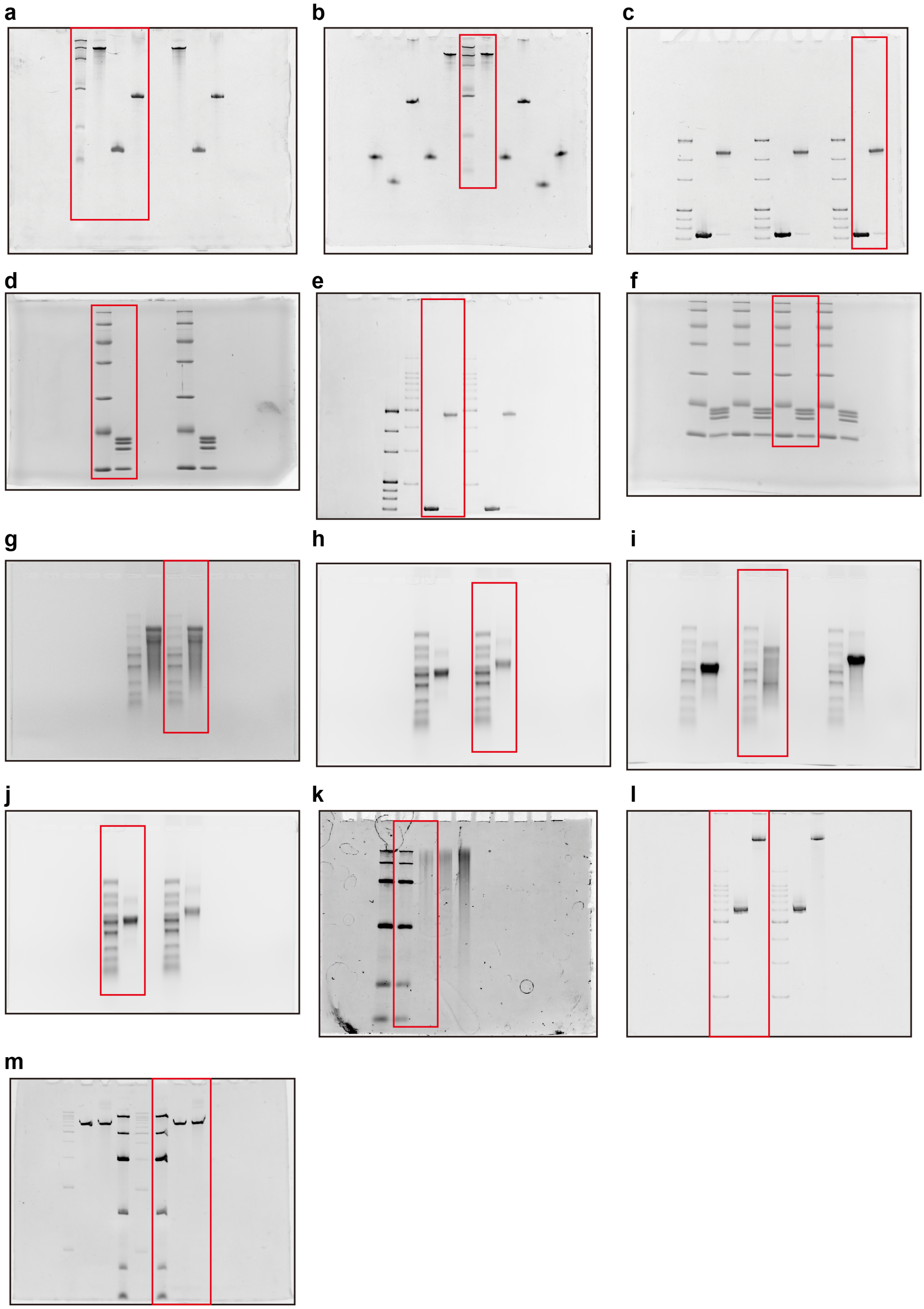

Supplementary Figure 5. Full images.

a, The full image of Fig. 3b. **b,** The full image of Supplementary Fig. 1a. **c,** The full image of Supplementary Fig. 1b. **d,** The full image of Supplementary Fig. 1c. **e,** The full image of Supplementary Fig. 1d. **f,** The full image of Supplementary Fig. 1e. **g,** The full image of Supplementary Fig. 2a. **h,** The full image of Supplementary Fig. 2b. **i,** The full image of

Supplementary Fig. 2c. **j,** The full image of Supplementary Fig. 2d. **k,** The full image of Supplementary Fig. 2e. **l,** The full image of Supplementary Fig. 3a. **m,** The full image of Supplementary Fig. 3b.

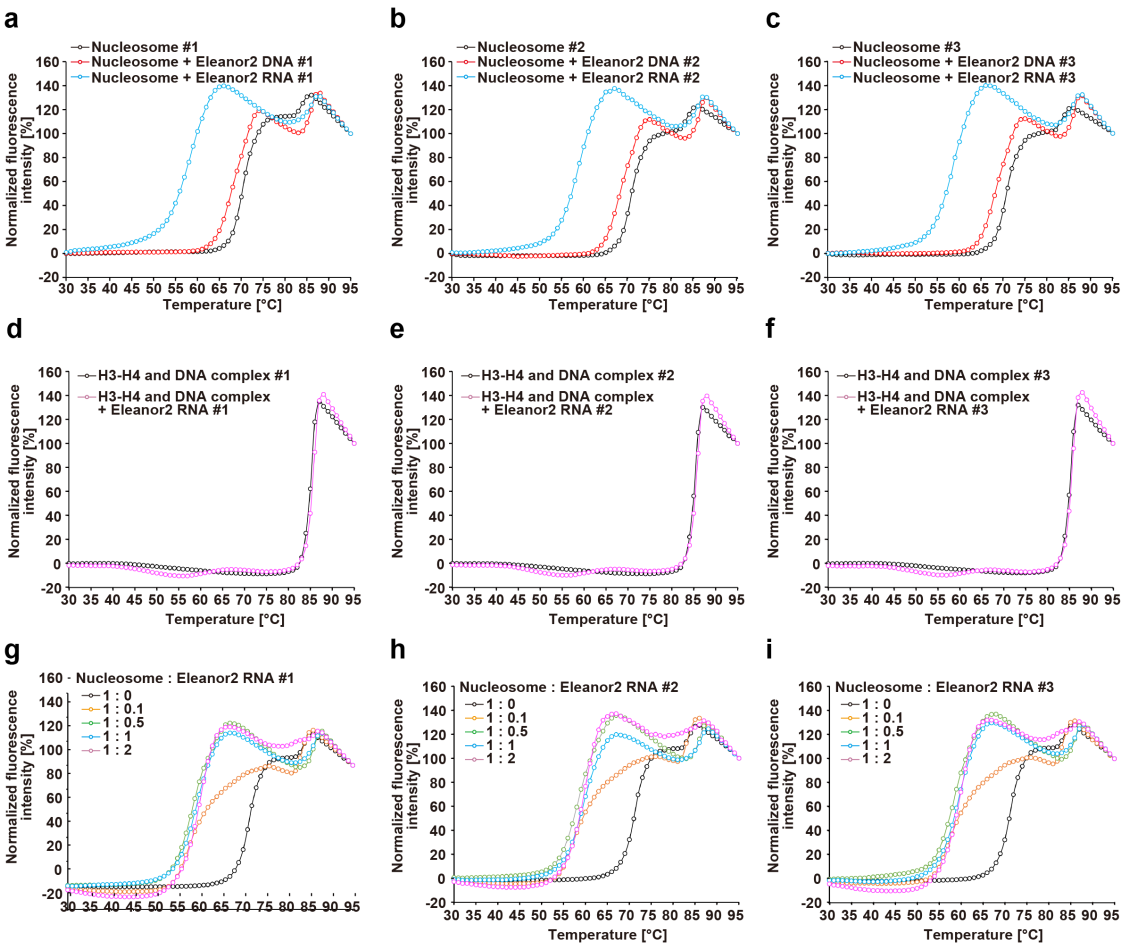

Supplementary Figure 6. The source data of the thermal stability assays presented in Fig. 2.

a-c, The source data for Fig. 2b. **d-f,** The source data for Fig. 2c. **g-i,** The source data for Fig. 2d.

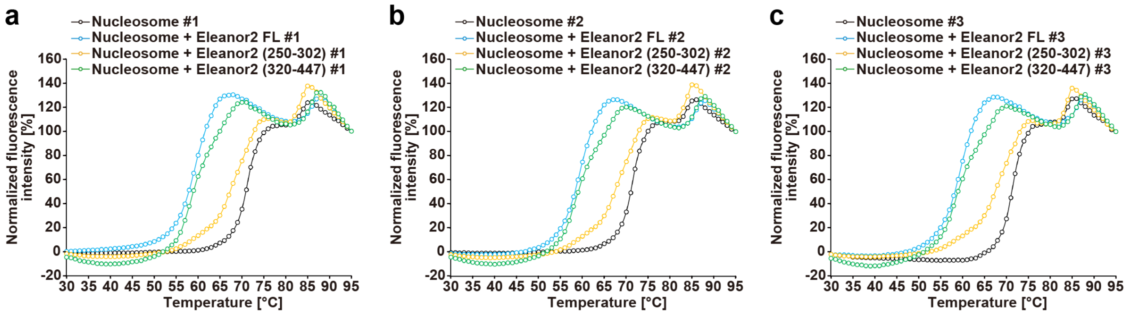

Supplementary Figure 7. The source data of the thermal stability assays presented in Fig. 3.

a-c, The source data for Fig. 3c.

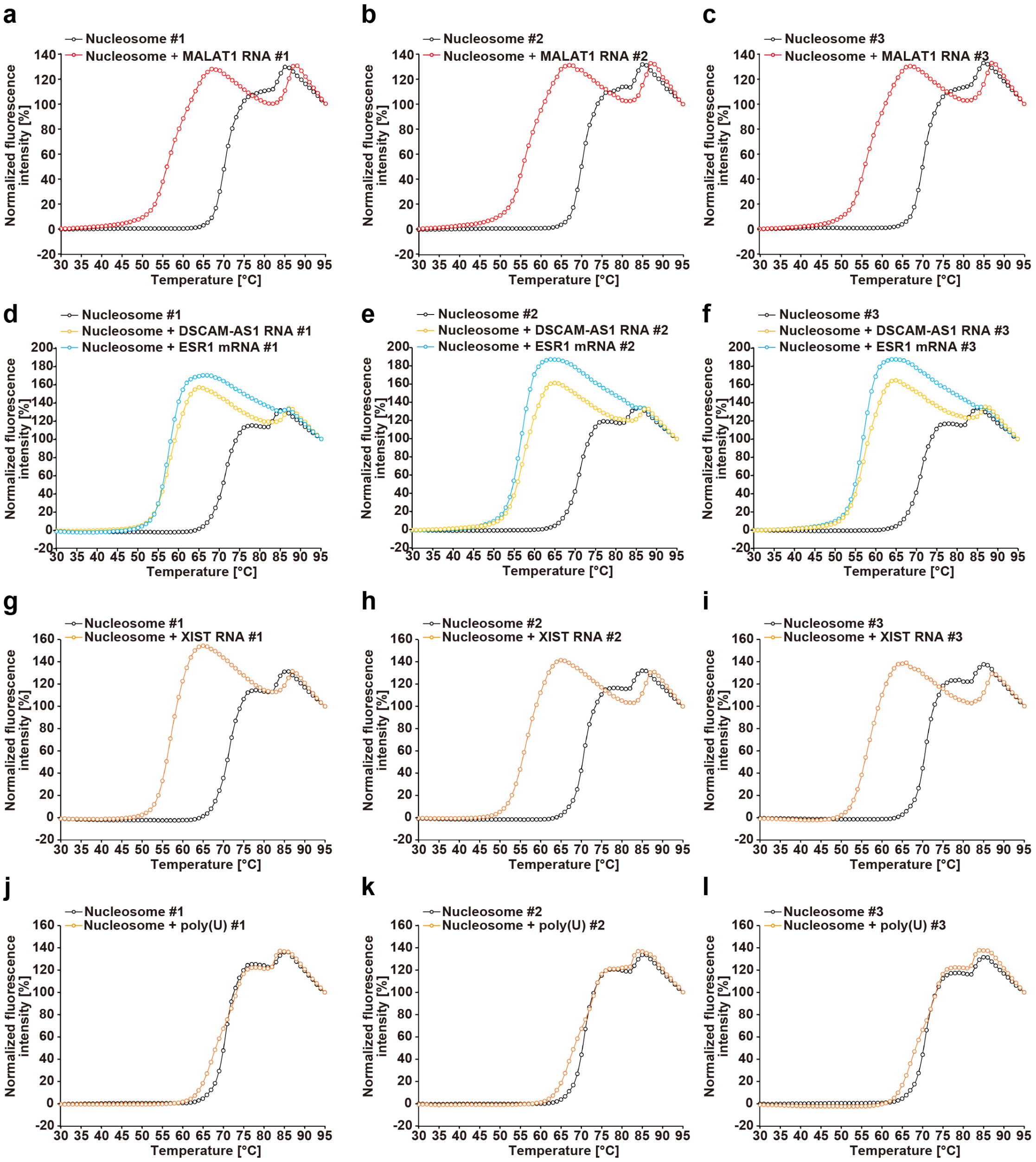

Supplementary Figure 8. The source data of the thermal stability assays presented in Fig. 4.

a-c, The source data for Fig. 4a. **d-f,** The source data for Fig. 4b and 4d. **g-i,** The source data for Fig. 4c. **j-l,** The source data for Fig. 4e.

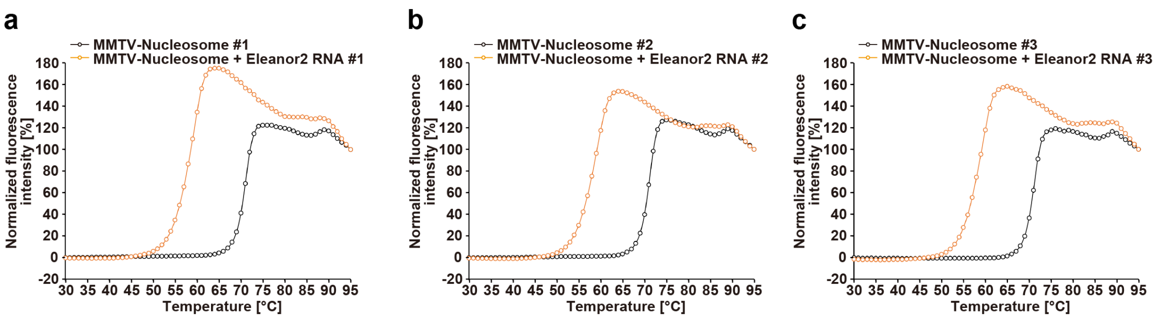

Supplementary Figure 9. The source data of the thermal stability assays presented in Supplementary Fig. 1.

a-c, The source data for Supplementary Fig. 1f.

SUPPLEMENTARY TABLES

Supplementary Table 1. Primer sets for qRT-PCR

name	sequence
**ESR1**	5'-CAGGCCAAATTCAGATAATCG-3'
	5'-TCCTTGGCAGATTCCATAGC-3'
**Eleanor2**	5'-CGCCTCTGAAGTTTGCATC-3'
	5'-GTCTGGGTGCCACTGTTTG-3'
**GAPDH**	5'-ACACCCACTCCTCCACCTTT-3'
	5'-TAGCCAAATTCGTTGTCATACC-3'

Supplementary Table 2. Primer sets for FAIRE-qPCR

name	sequence
**"A"**	5'-CGCCTCTGAAGTTTGCATC-3'
	5'-GTCTGGGTGCCACTGTTTG-3'
**"B"**	5'-TGAGCTATGCCTTTGTCCAG-3'
	5'-TTAAGAGCAAGCGTCCTTGG-3'
**"C"**	5'-GCCATCCAAGAAAGGTACCTTG-3'
	5'-CAGTGTGGGCTGCAGTTAAC-3'
**"D"**	5'-CACAGACGGTTTGGAGGGGT-3'
	5'-GGCATTTCTGTGCTTTGGAGGT-3'
