# Supplementary material for: Nucleosome destabilization by nuclear non-coding RNAs
Source: Commun Biol. 2020 Feb 11;3:60. doi: 10.1038/s42003-020-0784-9 (PMC7012929; doi:10.1038/s42003-020-0784-9)
Supplement: Supplementary file 2 — Description of Additional Supplementary Files [file 42003_2020_784_MOESM2_ESM.pdf]

Description of Additional Supplementary Files

File Name: Supplementary Data 1
Description: Data used for real-time PCR quantification in Figures 1c, 1f, 5a, and 5e.

File Name: Supplementary Data 2
Description: The RNA sequences employed for the thermal stability assay in Figure 4.
