# Supplementary material for: Nucleosome destabilization by nuclear non-coding RNAs
Source: Commun Biol. 2020 Feb 11;3:60. doi: 10.1038/s42003-020-0784-9 (PMC7012929; doi:10.1038/s42003-020-0784-9)
Supplement: Supplementary file 4 — Supplementary Data 2 [file 42003_2020_784_MOESM4_ESM.docx]

**Sequences of RNA transcribed *in vitro***

3,720-base MALAT1 containing GGG (derived from the T7 promoter) at the 5'-terminus and GAU (derived from digestion with *Eco*RV) at the 3'-terminus

GGGGGAAGACAGAAGUACGGGAAGGCGAAGAAAAGAAUAGAGAAGAUAGGGAAAUUAGAAGAUAAAAACAUACUUUUAGAAGAAAAAAGAUAAAUUUAAACCUGAAAAGUAGGAAGCAGAAGAAAAAAGACAAGCUAGGAAACAAAAAGCUAAGGGCAAAAUGUACAAACUUAGAAGAAAAUUGGAAGAUAGAAACAAGAUAGAAAAUGAAAAUAUUGUCAAGAGUUUCAGAUAGAAAAUGAAAAACAAGCUAAGACAAGUAUUGGAGAAGUAUAGAAGAUAGAAAAAUAUAAAGCCAAAAAUUGGAUAAAAUAGCACUGAAAAAAUGAGGAAAUUAUUGGUAACCAAUUUAUUUUAAAAGCCCAUCAAUUUAAUUUCUGGUGGUGCAGAAGUUAGAAGGUAAAGCUUGAGAAGAUGAGGGUGUUUACGUAGACCAGAACCAAUUUAGAAGAAUACUUGAAGCUAGAAGGGGAAGUUGGUUAAAAAUCACAUCAAAAAGCUACUAAAAGGACUGGUGUAAUUUAAAAAAAACUAAGGCAGAAGGCUUUUGGAAGAGUUAGAAGAAUUUGGAAGGCCUUAAAUAUAGUAGCUUAGUUUGAAAAAUGUGAAGGACUUUCGUAACGGAAGUAAUUCAAGAUCAAGAGUAAUUACCAACUUAAUGUUUUUGCAUUGGACUUUGAGUUAAGAUUAUUUUUUAAAUCCUGAGGACUAGCAUUAAUUGACAGCUGACCCAGGUGCUACACAGAAGUGGAUUCAGUGAAUCUAGGAAGACAGCAGCAGACAGGAUUCCAGGAACCAGUGUUUGAUGAAGCUAGGACUGAGGAGCAAGCGAGCAAGCAGCAGUUCGUGGUGAAGAUAGGAAAAGAGUCCAGGAGCCAGUGCGAUUUGGUGAAGGAAGCUAGGAAGAAGGAAGGAGCGCUAACGAUUUGGUGGUGAAGCUAGGAAAAAGGAUUCCAGGAAGGAGCGAGUGCAAUUUGGUGAUGAAGGUAGCAGGCGGCUUGGCUUGGCAACCACACGGAGGAGGCGAGCAGGCGUUGUGCGUAGAGGAUCCUAGACCAGCAUGCCAGUGUGCCAAGGCCACAGGGAAAGCGAGUGGUUGGUAAAAAUCCGUGAGGUCGGCAAUAUGUUGUUUUUCUGGAACUUACUUAUGGUAACCUUUUAUUUAUUUUCUAAUAUAAUGGGGGAGUUUCGUACUGAGGUGUAAAGGGAUUUAUAUGGGGACGUAGGCCGAUUUCCGGGUGUUGUAGGUUUCUCUUUUUCAGGCUUAUACUCAUGAAUCUUGUCUGAAGCUUUUGAGGGCAGACUGCCAAGUCCUGGAGAAAUAGUAGAUGGCAAGUUUGUGGGUUUUUUUUUUUUACACGAAUUUGAGGAAAACCAAAUGAAUUUGAUAGCCAAAUUGAGACAAUUUCAGCAAAUCUGUAAGCAGUUUGUAUGUUUAGUUGGGGUAAUGAAGUAUUUCAGUUUUGUGAAUAGAUGACCUGUUUUUACUUCCUCACCCUGAAUUCGUUUUGUAAAUGUAGAGUUUGGAUGUGUAACUGAGGCGGGGGGGAGUUUUCAGUAUUUUUUUUUGUGGGGGUGGGGGCAAAAUAUGUUUUCAGUUCUUUUUCCCUUAGGUCUGUCUAGAAUCCUAAAGGCAAAUGACUCAAGGUGUAACAGAAAACAAGAAAAUCCAAUAUCAGGAUAAUCAGACCACCACAGGUUUACAGUUUAUAGAAACUAGAGCAGUUCUCACGUUGAGGUCUGUGGAAGAGAUGUCCAUUGGAGAAAUGGCUGGUAGUUACUCUUUUUUCCCCCCACCCCCUUAAUCAGACUUUAAAAGUGCUUAACCCCUUAAACUUGUUAUUUUUUACUUGAAGCAUUUUGGGAUGGUCUUAACAGGGAAGAGAGAGGGUGGGGGAGAAAAUGUUUUUUUCUAAGAUUUUCCACAGAUGCUAUAGUACUAUUGACAAACUGGGUUAGAGAAGGAGUGUACCGCUGUGCUGUUGGCACGAACACCUUCAGGGACUGGAGCUGCUUUUAUCCUUGGAAGAGUAUUCCCAGUUGAAGCUGAAAAGUACAGCACAGUGCAGCUUUGGUUCAUAUUCAGUCAUCUCAGGAGAACUUCAGAAGAGCUUGAGUAGGCCAAAUGUUGAAGUUAAGUUUUCCAAUAAUGUGACUUCUUAAAAGUUUUAUUAAAGGGGAGGGGCAAAUAUUGGCAAUUAGUUGGCAGUGGCCUGUUACGGUUGGGAUUGGUGGGGUGGGUUUAGGUAAUUGUUUAGUUUAUGAUUGCAGAUAAACUCAUGCCAGAGAACUUAAAGUCUUAGAAUGGAAAAAGUAAAGAAAUAUCAACUUCCAAGUUGGCAAGUAACUCCCAAUGAUUUAGUUUUUUUCCCCCCAGUUUGAAUUGGGAAGCUGGGGGAAGUUAAAUAUGAGCCACUGGGUGUACCAGUGCAUUAAUUUGGGCAAGGAAAGUGUCAUAAUUUGAUACUGUAUCUGUUUUCCUUCAAAGUAUAGAGCUUUUGGGGAAGGAAAGUAUUGAACUGGGGGUUGGUCUGGCCUACUGGGCUGACAUUAACUACAAUUAUGGGAAAUGCAAAAGUUGUUUGGAUAUGGUAGUGUGUGGUUCUCUUUUGGAAUUUUUUUCAGGUGAUUUAAUAAUAAUUUAAAACUACUAUAGAAACUGCAGAGCAAAGGAAGUGGCUUAAUGAUCCUGAAGGGAUUUCUUCUGAUGGUAGCUUUUGUAUUAUCAAGUAAGAUUCUAUUUUCAGUUGUGUGUAAGCAAGUUUUUUUUUUUAGUGUAGGAGAAAUACUUUUCCAUUGUUUAACUGCAAAACAAGAUGUUAAGGUAUGCUUCAAAAAUUUUGUAAAUUGUUUAUUUUAAACUUAUCUGUUUGUAAAUUGUAACUGAUUAAGAAUUGUGAUAGUUCAGCUUGAAUGUCUCUUAGAGGGUGGGCUUUUGUUGAUGAGGGAGGGGAAACUUUUUUUUUUCUAUAGACUUUUUUCAGAUAACAUCUUCUGAGUCAUAACCAGCCUGGCAGUAUGAUGGCCUAGAUGCAGAGAAAACAGCUCCUUGGUGAAUUGAUAAGUAAAGGCAGAAAAGAUUAUAUGUCAUACCUCCAUUGGGGAAUAAGCAUAACCCUGAGAUUCUUACUACUGAUGAGAACAUUAUCUGCAUAUGCCAAAAAAUUUUAAGCAAAUGAAAGCUACCAAUUUAAAGUUACGGAAUCUACCAUUUUAAAGUUAAUUGCUUGUCAAGCUAUAACCACAAAAAUAAUGAAUUGAUGAGAAAUACAAUGAAGAGGCAAUGUCCAUCUCAAAAUACUGCUUUUACAAAAGCAGAAUAAAAGCGAAAAGAAAUGAAAAUGUUACACUACAUUAAUCCUGGAAUAAAAGAAGCCGAAAUAAAUGAGAGAUGAGUUGGGAUCAAGUGGAUUGAGGAGGCUGUGCUGUGUGCCAAUGUUUCGUUUGCCUCAGACAGGUAUCUCUUCGUUAUCAGAAGAGUUGCUUCAUUUCAUCUGGGAGCAGAAAACAGCAGGCAGCUGUUAACAGAUAAGUUUAACUUGCAUCUGCAGUAUUGCAUGUUAGGGAUAAGUGCUUAUUUUUAAGAGCUGUGGAGUUCUUAAAUAUCAACCAUGGCACUUUCUCCUGACCCCUUCCCUAGGGGAUUUCAGGAUUGAGAAAUUUUUCCAUCGAGCCUUUUUAAAAUUGUAGGACUUGUUCCUGUGGGCUUCAGUGAUGGAU

2,463-base DSCAM-AS1 containing GGG (derived from the T7 promoter) at the 5'-terminus and UCG (derived from digestion with *Nru*I) at the 3'-terminus

GGGAAUCCAAGUUACCUAAAAGGCUAAUUCCUAAUGAGAAGGAAAGGAAACAUACCAUAAAGUAAAAGUGCAAACAAUUGCAAAACAGAGGCAAACAGUGUCAGUUGGAUAUACGUAAGCGCUGAUGUAAAGAGAGAUUGGAAAUGAUAUAUCUGGCUGGGCAGGUCACUCAUGCCUGUAAUCCCAGCACUUUGGGAGGCUGAGGCAGGCAGAUCACCUAAGGCCAGGAAUUCGACACCAGCCUGGCCAACGUGGCAAAACCCGUCUCUACUAAAAAUACAAAAAUUAGCCGGGCGUGGUGGUGUGCGCCUGGAAUCCCAGCUACCCAGGAGGCUGAGGCAGGAGAAAUGCUGGAACCCGGGAGGCAGAGGCUGCAGUGAGCUGAGAUCAUGCCACUACUGCACUCCAGCAUGGGUGACACAGCAAGACUCCCUCUAAAAAAAGAAAAAAAGAAAAGAAAAGAAAAGAAAAUGAUAUAUCCAUGAUGAAUUAAAAUGGAGUGGAACCCACUGAUGGUAUGCAGCUGAUAAGACGCUAUAGAGAAAUGAUAUCCGGACACAUGGUGAGACUUUUUCUCCUCUGUAAAUCUCAAUUCUUGUGAUUCUUUCAAAGGGAAAGCCACAGAAGGUACCAGUUAUCCACUCACUGACUUAGGUGCCUCCACUAGAAUUCUCAGCAGGUUUUUGCAGAAGUAAGUUAUCUUCACUAUUGCUCUAACACUUCUUAGUCUCCUUUUUCUUUCUUUUCUUUUUUUUUAGACAAAGGAAAAGUAAACCGCUGGUUUAGAGUCUUUGGAAGGCAAUUGUAUCUGACUAGAAUUUAAUAUUAUUGUUUUUCUUUCAUUGUAUUGCAUUUAUCUGUAUGGCUAGUUAAACUGAUUUUCCAUUUAAAAGUAGUAAUGCAUUUUUUUAUAAUUACAUUGUUUUAAGUAAACAAGAAUUAUUCAAAGGACAAAAACACUAGCACAGAUGGCAUUCAAUGAUUGCAGAUAUCAUAAAGAUAGUUCAUGAAUGACAAAGAUUUGAGAAGUACUGUGUUCACGAUGCUUCCUGGAAGAGGUGGGUUAUACAGUGUUUCUUAGAGAAAGGGAUAGGGGUCUACUGCUAGGAGGGAAAGAUGAAGCUACUUAGGGGGACUAGGAAACACGCAGUUGGCCUGCCCUCUGCUUGGAGAUCACAGCCAAGGAAACACAGGAGAGGCACCCCCUGGAGGAGCUCCGUGGGCUGCAGGGCUGACGUGGCCCAGGUAAGUGGGAGUUCAGGGAGGCCCACAGGUUGGUGCUAGGAUAGUGGCAGAUGAUUUUCAUUGUCAGACUGUGAGACAAAAGGUAGACAUAAGGGAGGCAGAGGUGAAAUCGGGAUGGUUAAAACAGAGAAUGCUGAGAGCAGAGCAAGGCAGUUGCUAAAGCUGUUACUAAAACUGGACAGCAGAAGGCCUGGCGUGGUGGCUCACAGCUGUAAUCCCAGCACUUUGGGAGGCCUAGGUGGGUGGAUCAUCAGAGGUCAGGAGUUAGAGACUAGCCUGACCAACAUGGUGAAACCCCAUCUCUACUAAAAAUACAAAGUUAGCCAGGCAUGGUGGCCCAUGCCUGUAAUCCCAGCUACUCGGGAGGCUGAGGCAGGAGAAUUGCUUGAAUUUGGGAGGUGGAGGUUGCGGUGAGCUGAGAUCGCACCAUUGCUCUCCAGCCUGGGCAACAAGAGCGAAACCCCAUCUCAAAACCACAACAACAACAACAGGACAACAGAGAUGGACGACGGAUCGGGAAAGCCAACCAGACAGCGUGAGGCCAGGACGGAAAGAGGCACAGGGAGCUCUGCUCAGUGUCGCUACAGGGGAUCUCUCAGGCUCACAACGGGCCACUCCUCUAGGGAAGUUCUGGUCUCAUCAUGAUCCUUGUUUGGUCUCACUCCCCAUGUCCUUCUCUGUCCCUCCUCCAACUGCCAUUUAUUUAUUUAACUGAAAAAGUACCAAUCACCCACAUAGGCAUGACAUACUCAUCCAUGUACCCAUUUCUUAAAAUUGAUCAUUGUUAACAUUUGGUGUAAUUUGCUUUAUUUAUUUUUAAUGAAAUAAAUAAAACUUUACAGAAAAUGCUUUAUUUUUCUCUUUGUUCCCUCCCCAUCCUAUAUUUUUCUCCUAAAAAACCCUAUUAUCAGAAAUAUUAGUGUGUAUUCCCAGUUUCGACUUUUUAUUUUAUUACACACACACUCCCACACAUAGCUGUGACAAUGAACUUCACAUAGUAUGGUUCUGUAUUUUCUCUUUGUUUUUCAAACUUACAUAAGUAUUUUACUAUUUCUCAUGGAAAAACUCACUUUUCCCAUCCAACGUUAUGUUUCCUUAAGAUCUCUCUAUGUUGAUAUAAAGAAAUCUAGUUCAUUCUUUUUAAUGAAAAUAAAGUAUAUUUUAUGAAUGUAACUCAUGCUAACCAUGGCAAUAAAAGCUCCAUCAAGCAUGCAUUCG

3,037-base XIST containing GGG (derived from the T7 promoter) at the 5'-terminus and UCG (derived from digestion with *Nru*I) at the 3'-terminus

GGGCCUUCAGUUCUUAAAGCGCUGCAAUUCGCUGCUGCAGCCAUAUUUCUUACUCUCUCGGGGCUGGAAGCUUCCUGACUGAAGAUCUCUCUGCACUUGGGGUUCUUUCUAGAACAUUUUCUAGUCCCCCAACACCCUUUAUGGCGUAUUUCUUUAAAAAAAUCACCUAAAUUCCAUAAAAUAUUUUUUUAAAUUCUAUACUUUCUCCUAGUGUCUUCUUGACACGUCCUCCAUAUUUUUUUAAAGAAAGUAUUUGGAAUAUUUUGAGGCAAUUUUUAAUAUUUAAGGAAUUUUUCUUUGGAAUCAUUUUUGGUUGACAUCUCUGUUUUUUGUGGAUCAGUUUUUUACUCUUCCACUCUCUUUUCUAUAUUUUGCCCAUCGGGGCUGCGGAUACCUGGUUUUAUUAUUUUUUCUUUGCCCAACGGGGCCGUGGAUACCUGCCUUUUAAUUCUUUUUUAUUCGCCCAUCGGGGCCGCGGAUACCUGCUUUUUAUUUUUUUUUCCUUAGCCCAUCGGGGUAUCGGAUACCUGCUGAUUCCCUUCCCCUCUGAACCCCCAACACUCUGGCCCAUCGGGGUGACGGAUAUCUGCUUUUUAAAAAUUUUCUUUUUUUGGCCCAUCGGGGCUUCGGAUACCUGCUUUUUUUUUUUUAUUUUUCCUUGCCCAUCGGGGCCUCGGAUACCUGCUUUAAUUUUUGUUUUUCUGGCCCAUCGGGGCCGCGGAUACCUGCUUUGAUUUUUUUUUUUCAUCGCCCAUCGGUGCUUUUUAUGGAUGAAAAAAUGUUGGUUUUGUGGGUUGUUGCACUCUCUGGAAUAUCUACACUUUUUUUUGCUGCUGAUCAUUUGGUGGUGUGUGAGUGUACCUACCGCUUUGGCAGAGAAUGACUCUGCAGUUAAGCUAAGGGCGUGUUCAGAUUGUGGAGGAAAAGUGGCCGCCAUUUUAGACUUGCCGCAUAACUCGGCUUAGGGCUAGUCGUUUGUGCUAAGUUAAACUAGGGAGGCAAGAUGGAUGAUAGCAGGUCAGGCAGAGGAAGUCAUGUGCAUUGCAUGAGCUAAACCUAUCUGAAUGAAUUGAUUUGGGGCUUGUUAGGAGCUUUGCGUGAUUGUUGUAUCGGGAGGCAGUAAGAAUCAUCUUUUAUCAGUACAAGGGACUAGUUAAAAAUGGAAGGUUAGGAAAGACUAAGGUGCAGGGCUUAAAAUGGCGAUUUUGACAUUGCGGCAUUGCUCAGCAUGGCGGGCUGUGCUUUGUUAGGUUGUCCAAAAUGGCGGAUCCAGUUCUGUCGCAGUGUUCAAGUGGCGGGAAGGCCACAUCAUGAUGGGCGAGGCUUUGUUAAGUGGUUAGCAUGGUGGUGGACAUGUGCGGUCACACAGGAAAAGAUGGCGGCUGAAGGUCUUGCCGCAGUGUAAAACAUGGCGGGCCUCUUUGUCUUUGCUGUGUGCUUUUCGUGUUGGGUUUUGCCGCAGGGACAAUAUGGCAGGCGUUGUCAUAUGUAUAUCAUGGCUUUUGUCACGUGGACAUCAUGGCGGGCUUGCCGCAUUGUUAAAGAUGGCGGGUUUUGCCGCCUAGUGCCACGCAGAGCGGGAGAAAAGGUGGGAUGGACAGUGCUGGAUUGCUGCAUAACCCAACCAAUUAGAAAUGGGGGUGGAAUUGAUCACAGCCAAUUAGAGCAGAAGAUGGAAUUAGACUGAUGACACACUGUCCAGCUACUCAGCGAAGACCUGGGUGAAUUAGCAUGGCACUUCGCAGCUGUCUUUAGCCAGUCAGGAGAAAGAAGUGGAGGGGCCACGUGUAUGUCUCCCAGUGGGCGGUACACCAGGUGUUUUCAAGGUCUUUUCAAGGACAUUUAGCCUUUCCACCUCUGUCCCCUCUUAUUUGUCCCCUCCUGUCCAGUGCUGCCUCUUGCAGUGCUGGAUAUCUGGCUGUGUGGUCUGAACCUCCCUCCAUUCCUCUGUAUUGGUGCCUCACCUAAGGCUAAGUAUACCUCCCCCCCCACCCCCCAACCCCCCCAACUCCCCACCCCCCUACCCCCCUACCCCCCUACCCCCCUCUGGUCUGCCCUGCACUGCACUGUUGCCAUGGGCAGUGCUCCAGGCCUGCUUGGUGUGGACAUGGUGGUGAGCCGUGGCAAGGACCAGAAUGGAUCACAGAUGAUCGUUGGCCAACAGGUGGCAGAAGAGGAAUUCCUGCCUUCCUCAAGAGGAACACCUACCCCUUGGCUAAUGCUGGGGUCGGAUUUUGAUUUAUAUUUAUCUUUUGGAUGUCAGUCAUACAGUCUGAUUUUGUGGUUUGCUAGUGUUUGAAUUUAAGUCUUAAGUGACUAUUAUAGAAAUGUAUUAAGAGGCUUUAUUUGUAGAAUUCACUUUAAUUACAUUUAAUGAGUUUUUGUUUUGAGUUCCUUAAAAUUCCUUAAAGUUUUUAGCUUCUCAUUACAAAUUCCUUAACCUUUUUUUGGCAGUAGAUAGUCAAAGUCAAAUCAUUUCUAAUGUUUUAAAAAUGUGCUGGUCAUUUUCUUUGAAAUUGACUUAACUAUUUUCCUUUGAAGAGUCUGUAGCACAGAAACAGUAAAAAAUUUAACUUCAUGACCUAAUGUAAAAAAGAGUGUUUGAAGGUUUACACAGGUCCAGGCCUUGCUUUGUUCCCAUCCUUGAUGCUGCACUAAUUGACUAAUCACCUACUUAUCAGACAGGAAACUUGAAUUGCUGUGGUCUGGUGUCCUCUAUUCAGACUUAUUAUAUUGGAGUAUUUCAAUUUUUCGUUGUAUCCUGCCUGCCUAGCAUCCAGUUCCUCCCCAGCCCUGCUCCCAGCAAACCCCUAGUCUAGCCCCAGCCCUACUCCCACCCCGCCCCAGCCCUGCCCCAGCCCCAGUCCCCUAACCCCCCAGCCCUAGCCCCAGUCCCAGUCCUAGUUCCUCAGUCCCGCCCAGCUUCUCUCGAAAGUCACUCUAAUUUUCAUUGAUUCAGUGCUCAAAAUAAGUUGUCCAUUGCUUAUCCUAUUAUACUGGGAUAUUCCGUUUACCCUUGGCAUUGCUGAUCUUCUCG
