# Supplementary material for: Nucleosome destabilization by nuclear non-coding RNAs
Source: Commun Biol. 2020 Feb 11;3:60. doi: 10.1038/s42003-020-0784-9 (PMC7012929; doi:10.1038/s42003-020-0784-9)
Supplement: Supplementary file 5 — Reporting Summary [file 42003_2020_784_MOESM5_ESM.pdf]

nature research

Corresponding author(s): Hitoshi Kurumizaka, Noriko Saitoh

Last updated by author(s): Dec 27, 2019

Reporting Summary

Nature Research wishes to improve the reproducibility of the work that we publish. This form provides structure for consistency and transparency in reporting. For further information on Nature Research policies, see Authors & Referees and the Editorial Policy Checklist.

Statistics

For all statistical analyses, confirm that the following items are present in the figure legend, table legend, main text, or Methods section.

n/a	Confirmed	
☐	☒	The exact sample size (*n*) for each experimental group/condition, given as a discrete number and unit of measurement
☐	☒	A statement on whether measurements were taken from distinct samples or whether the same sample was measured repeatedly
☐	☒	The statistical test(s) used AND whether they are one- or two-sided<br>*Only common tests should be described solely by name; describe more complex techniques in the Methods section.*
☒	☐	A description of all covariates tested
☐	☒	A description of any assumptions or corrections, such as tests of normality and adjustment for multiple comparisons
☐	☒	A full description of the statistical parameters including central tendency (e.g. means) or other basic estimates (e.g. regression coefficient) AND variation (e.g. standard deviation) or associated estimates of uncertainty (e.g. confidence intervals)
☐	☒	For null hypothesis testing, the test statistic (e.g. *F*, *t*, *r*) with confidence intervals, effect sizes, degrees of freedom and *P* value noted<br>*Give P values as exact values whenever suitable.*
☒	☐	For Bayesian analysis, information on the choice of priors and Markov chain Monte Carlo settings
☒	☐	For hierarchical and complex designs, identification of the appropriate level for tests and full reporting of outcomes
☒	☐	Estimates of effect sizes (e.g. Cohen's *d*, Pearson's *r*), indicating how they were calculated

Our web collection on statistics for biologists contains articles on many of the points above.

Software and code

Policy information about availability of computer code

Data collection	Sequencing for ATAC-seq was performed using Illumina Mi-seq.
Data analysis	For RNA secondary structure prediction, CentroidFold (Hamada et al., Bioinformatics 2009) was used.<br>For FAIRE-seq analyses, BWA (v0.7.12), MarkDuplicates (Picard v1.136), and Integrative Genomics Viewer (v. 2.3.68) tools were used. For RNA-seq analyses, Tophat (v1.4.1) and Cufflinks (v1.3.0) as previously described in Tomita et al., Nat commun 2015 were used. For ATAC-seq analyses, Bowtie2 (v. 2.3.4.3), MarkDuplicates (Picard v1.136), Integrative Genomics Viewer (v. 2.3.68) tools, and BamCompare of Deep tools (Ramírez et al., Nucleic Acids Res. 2014) were used. Details are included in the Method section.

For manuscripts utilizing custom algorithms or software that are central to the research but not yet described in published literature, software must be made available to editors/reviewers. We strongly encourage code deposition in a community repository (e.g. GitHub). See the Nature Research guidelines for submitting code & software for further information.

Data

Policy information about availability of data

All manuscripts must include a data availability statement. This statement should provide the following information, where applicable:

- Accession codes, unique identifiers, or web links for publicly available datasets
- A list of figures that have associated raw data
- A description of any restrictions on data availability

ATAC-seq raw data are available in the DDBJ Sequenced Read Archive, under the accession number DRA008967.

The data that support the findings of this study are available from the corresponding authors upon reasonable request.

The raw full image data of Fig. 3b was shown in Supplementary Figure 5a. The raw full image data of Supplementary Figure 1a, b, c, d and e were shown in Supplementary Figure 5b, c, d, e and f, respectively. The raw full image data of Supplementary Figure 2a, b, c, d and e were shown in Supplementary Figure 5g, h, i, j

and k, respectively. The raw full image data of Supplementary Figure 3a and b were shown in Supplementary Figure 5l and m, respectively.

Field-specific reporting

Please select the one below that is the best fit for your research. If you are not sure, read the appropriate sections before making your selection.

☒ Life sciences ☐ Behavioural & social sciences ☐ Ecological, evolutionary & environmental sciences

For a reference copy of the document with all sections, see nature.com/documents/nr-reporting-summary-flat.pdf

Life sciences study design

All studies must disclose on these points even when the disclosure is negative.

Sample size	Sample size was employed as experimental repeats, by which error values of each experiment were properly obtained.
Data exclusions	No data were excluded from analyses.
Replication	The reproducibility for all biochemical analyses was confirmed by three independent experiments. The reproducibility for cell analysis was confirmed by three independent experiments.
Randomization	This was not relevant to our study.
Blinding	This was not relevant to our study.

Reporting for specific materials, systems and methods

We require information from authors about some types of materials, experimental systems and methods used in many studies. Here, indicate whether each material, system or method listed is relevant to your study. If you are not sure if a list item applies to your research, read the appropriate section before selecting a response.

Materials & experimental systems

n/a	Involved in the study	
☒	☐	Antibodies
☐	☒	Eukaryotic cell lines
☒	☐	Palaeontology
☒	☐	Animals and other organisms
☒	☐	Human research participants
☒	☐	Clinical data

Methods

n/a	Involved in the study	
☒	☐	ChIP-seq
☒	☐	Flow cytometry
☒	☐	MRI-based neuroimaging

Eukaryotic cell lines

Policy information about cell lines

Cell line source(s)	MCF10A cells (purchased from ATCC), MCF7 cells (purchased from ATCC) and LTED cells were established by culturing MCF7 cells in estrogen deprivation culture medium
Authentication	All cells were not authenticated.
Mycoplasma contamination	All cell lines were tested negative for mycoplasma contamination.
Commonly misidentified lines (See ICLAC register)	Cell line used is not in the ICLAC database.
